# Comparison of Intra-Examiner Agreement in the In Vivo Diagnosis of Carious Lesions With and Without Loupes Using the ICDAS II System

**DOI:** 10.4317/jced.63550

**Published:** 2026-01-28

**Authors:** Manuel De Feo-Tolosa, Maria del Rosario Garcillán-Izquierdo, Nicole Rodrigues, María Victoria Mateos-Moreno

**Affiliations:** 1 Department of Clinical Dental Specialties, Dentistry Faculty, Universidad Complutense, Madrid, Spain; 2 Department of Conservative and Prosthetic Dentistry, Dentistry Faculty, Universidad Complutense, Madrid, Spain

## Abstract

**Background:**

Accurate early caries detection remains a major challenge in minimally invasive dentistry. While visual inspection remains the clinical gold standard, magnification loupes are increasingly used in dental practice. However, their diagnostic reliability under real clinical conditions remains unclear. Objective: To evaluate intra-examiner agreement in the diagnosis of occlusal carious lesions using the ICDAS II system with and without 3.5x magnification loupes.

**Material and Methods:**

A cross-sectional in vivo study was conducted on 143 teeth (572 measurements) from 15 patients. Each occlusal surface was examined twice with and twice without loupes, randomized by sequence and separated by at least seven days. The examiner was calibrated and blinded to prior results. Weighted Cohen's Kappa coefficients, sensitivity, and specificity were calculated for each lesion type.

**Results:**

Concordance between methods was moderate (weighted Kappa = 0.56; 95% CI: 0.43-0.69). Intra-examiner reproducibility was high for both loupes (Kappa = 0.88) and unaided inspection (Kappa = 0.81). Sensitivity and specificity were highest for incipient and severe lesions, while low prevalence limited estimates for moderate lesions. Overall diagnostic accuracy for the magnified method was 0.85 (95% CI: 0.80-0.89).

**Conclusions:**

Magnification loupes provide comparable diagnostic accuracy to unaided visual inspection, with improved sensitivity for early lesions. Their systematic use may enhance diagnostic consistency and clinical training in preventive and minimally invasive dentistry.

## Introduction

The current management of caries considers new therapeutic strategies that require proper diagnosis (1). The clinical decision on how to approach each carious lesion will depend on the patient's risk level, the stage of the lesion, its activity, cavitation or not, and accessibility for cleaning (2). Therefore, diagnosis becomes highly important. Various methods and devices are available that not only assist in detecting lesions but also in their subsequent monitoring (3 , 4). On occlusal surfaces, visual examination remains the gold standard; however, the human eye lacks the acuity to see most pits and fissures (5). Among the complementary diagnostic systems, we find photon-based detection technologies: transillumination (FOTI and DIFOTI), fluorescence-based detection (DIAGNOdent Pen®), and the combination of fluorescence and images captured with a camera (Soprolife® and Soprocare®) (6). Contradictory results are still found in the literature regarding the effectiveness of dental loupes, and less than 3% of general dentists combine visual inspection, radiography, and one of the complementary systems mentioned above (7). The use of magnification in various fields of dentistry has many benefits (8 - 12); however, there is no conclusive literature on whether it is really effective for diagnosing carious lesions (10 - 13) or if it might even lead to overtreatment (10). Our main objective is to compare the intra-examiner concordance in caries diagnosis using ICDAS II with 3.5x magnifying loupes and without them on occlusal surfaces. We will compare the diagnostic concordance with loupes against the gold standard: caries diagnosis without loupes, by visual inspection. As a secondary objective, specificity and sensitivity indices will be obtained and analyzed according to lesion type: surfaces without lesions, initial, moderate, and severe stages.

## Material and Methods

**Figure 1:**
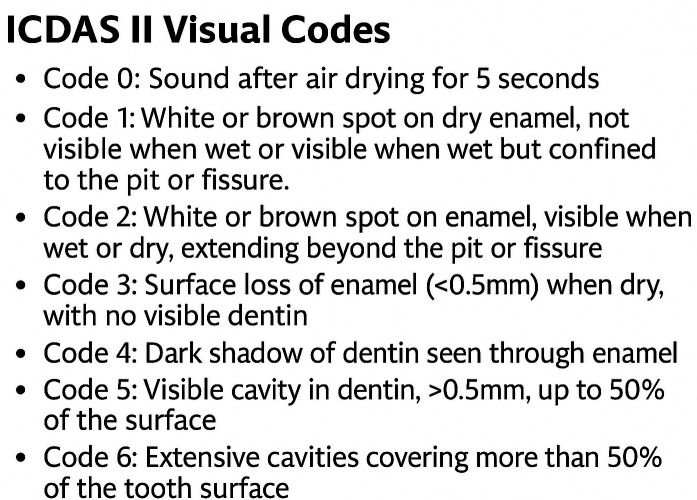
ICDASII Visual Codes.

1. Study Design This is a descriptive cross-sectional study conducted in a private dental clinic in Las Rozas, Madrid, with the standardized center code REGCESS (CCN) 1328027260. 2. Sample Size The G-Power software was used to calculate the sample size. The assumptions for the calculation were a type 1- error of 0.05 and a power of 80%. Another assumption was that the expected effect size in the difference in caries diagnosis with and without loupes is at least 0.2 (14). Based on this calculation, the minimum sample size was 138 occlusal surfaces. 3. Sample Inclusion criteria: occlusal surfaces of permanent teeth, occlusal surfaces without restorations, occlusal surfaces without sealants, and surfaces of fully erupted teeth. Exclusion criteria: surfaces with enamel development defects, surfaces of teeth from patients who do not sign the informed consent, and surfaces of teeth from patients who attend less than four visits. The sampling is non-probabilistic, of consecutive cases as patients come to the dental clinic for a dental check-up. If a patient needed treatments that required a minimum of four visits, they were included in the study. Measurements were taken at the beginning of each appointment. 4. Researchers The researchers in this study are part of the teaching team of the Preventive and Community Dentistry course at Complutense University of Madrid UCM, with extensive experience in using ICDAS II, and some are accredited as trainers in the ICDAS II system by instructors from the University of Leeds. The examiner has been using 3.5x loupes in routine practice for seven years and has eight years of clinical experience with ICDAS II, attending multiple calibration courses since 2017 led by official calibrators. Additionally, the examiner will undergo a new intra-examiner calibration update prior to the study measurements. Using the ICDAS II method (shown in Fig. 1), dental surfaces are diagnosed after prior prophylaxis (BESTDENT® prophylaxis paste and a rotary prophylaxis brush for contra-angle), before and after drying, meaning that the surfaces are inspected wet and dry. Inspection is visual and tactile, using a rounded-tip WHO probe (CP 11.5B SILVER HU FREDY®).

1

If multiple carious lesions are present on the same surface, the one with the highest value is scored. 5. Interventions An intraoral visual diagnosis will be performed on all occlusal surfaces of each patient. Each surface will be measured twice with and twice without loupes, with a minimum interval of seven days and a maximum of thirty. Diagnosis will be performed with standard dental chair lighting (Kavo®, Estetica E3, Germany), rhodium mirrors, and 3.5x fixed prismatic magnification loupes (Orascoptic®, Middleton, USA) custom-fitted for the examiner. Measurements will be made at a working distance of 350mm. Measurements will be recorded by the assistant facing away from the dentist on the printed data sheet (Appendix 1), noting the date of measurement. Thus, the assistant will be blinded as to whether the measurement was taken with or without loupes. At the end of the measurement, the dentist notes the date and whether the measurement was taken with or without loupes on the back of the sheet. A coin will be tossed before the first measurement to randomize the starting method. If heads, the examination will begin with the two measurements using loupes; if tails, the measurements without loupes will be conducted first. During the second, third, and fourth examination of the dental surfaces, the examiner will be blinded to the results obtained on the first day, with the dental assistant retrieving the patient's sheet to record the results. 2.6 Variables Since both variables are qualitative in a frequency study, the tooth surface is considered the fixed variable, and the ICDAS code is the random variable, which is a type of ordinal qualitative variable. 7. Ethics, Records, and Consent This study protocol was reviewed and approved by Ethics Committee of the San Carlos Clinical Hospital, Community of Madrid, approval number: 24/399-E (Appendix 4) (Supplement 1) http://www.medicinaoral.com/medoralfree01/aop/jced_63550_s01. Information sheet and written informed consent was provided to all patients (Appendix 2) (Supplement 1) http://www.medicinaoral.com/medoralfree01/aop/jced_63550_s01. 8. Statistical Analysis A statistical significance level of p &lt;0.05 and a power of 80% will be accepted. A two-tailed test will be performed. The intra-examiner Cohen's Kappa index was measured with a chi-square table for the total surfaces, comparing the total ICDAS codes between measurements with and without loupes to obtain the result related to the main objective. Cohen's Kappa index was also measured between measurements taken on different days with only loupes and only without loupes, to compare the precision of both methods. Similarly, concordance will be compared among incipient (ICDAS 1 and 2), moderate (ICDAS 3 and 4), and advanced lesions (ICDAS 5 and 6). Sensitivity and specificity of the diagnosis with loupes were measured, taking visual inspection without magnification as the gold standard. 9. Study Difficulties and Limitations The objective of this study is to measure the accuracy of diagnosis with dental loupes; it is not possible to determine the exact accuracy of carious lesion measurements with loupes. This is because the gold standard in our study is the visual inspection of the tooth surface, without histological or radiological information. For the same reason, we do not consider results from different examiners to be a priority, although it would be recommended. 10. Applicability and Practical Utility of Results Knowing the diagnostic accuracy with loupes will help confirm whether magnification is justified in clinical practice and research, where calibration and precision are key for data recording.

## Results

**Figure 2:**
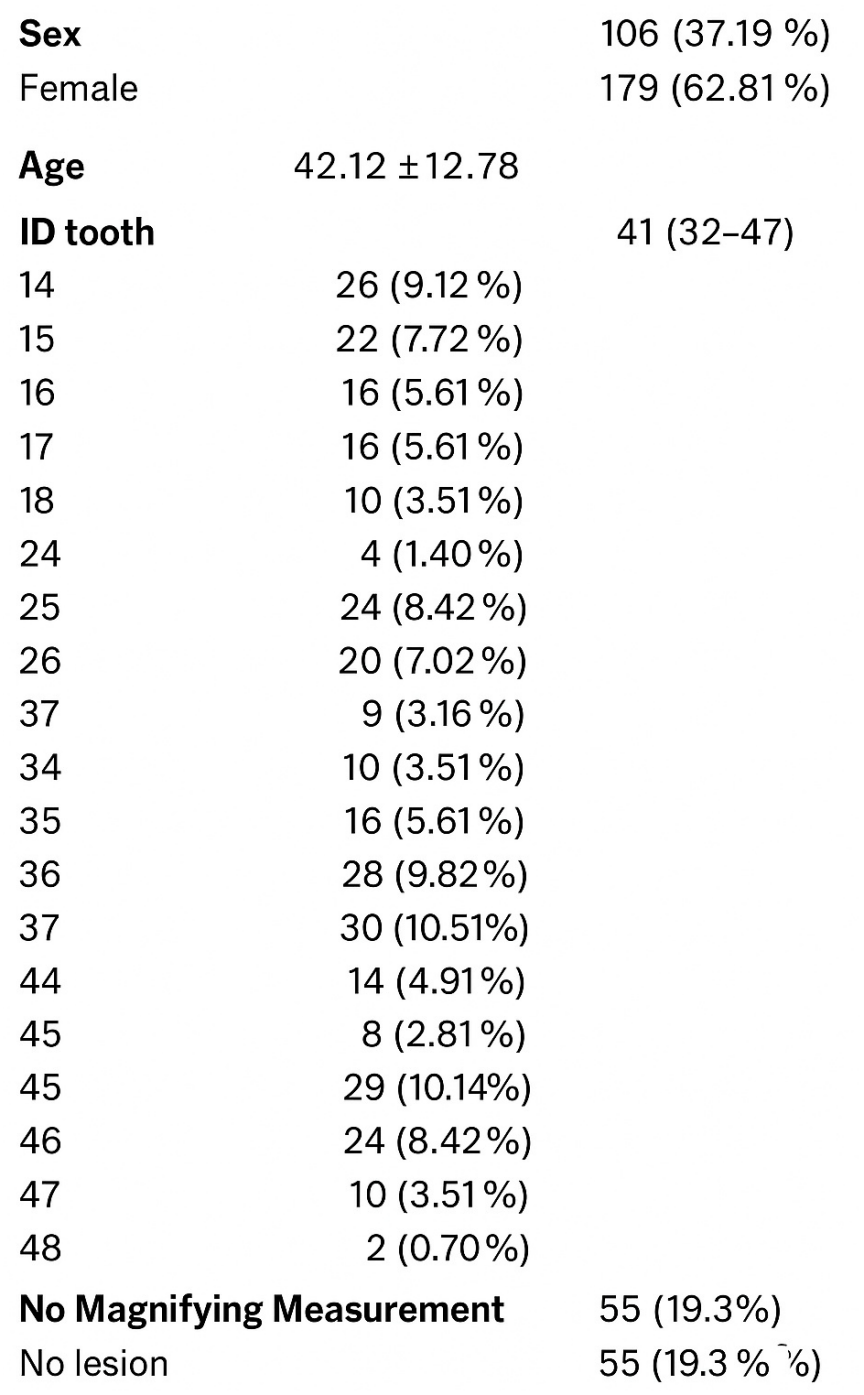
Descriptive Results.

A sample of 143 teeth from 15 patients was analyzed (572 measurements in total). The table below shows the descriptive analysis of the variables considered in the study (shown in Fig. 2).

2

**Figure 3:**
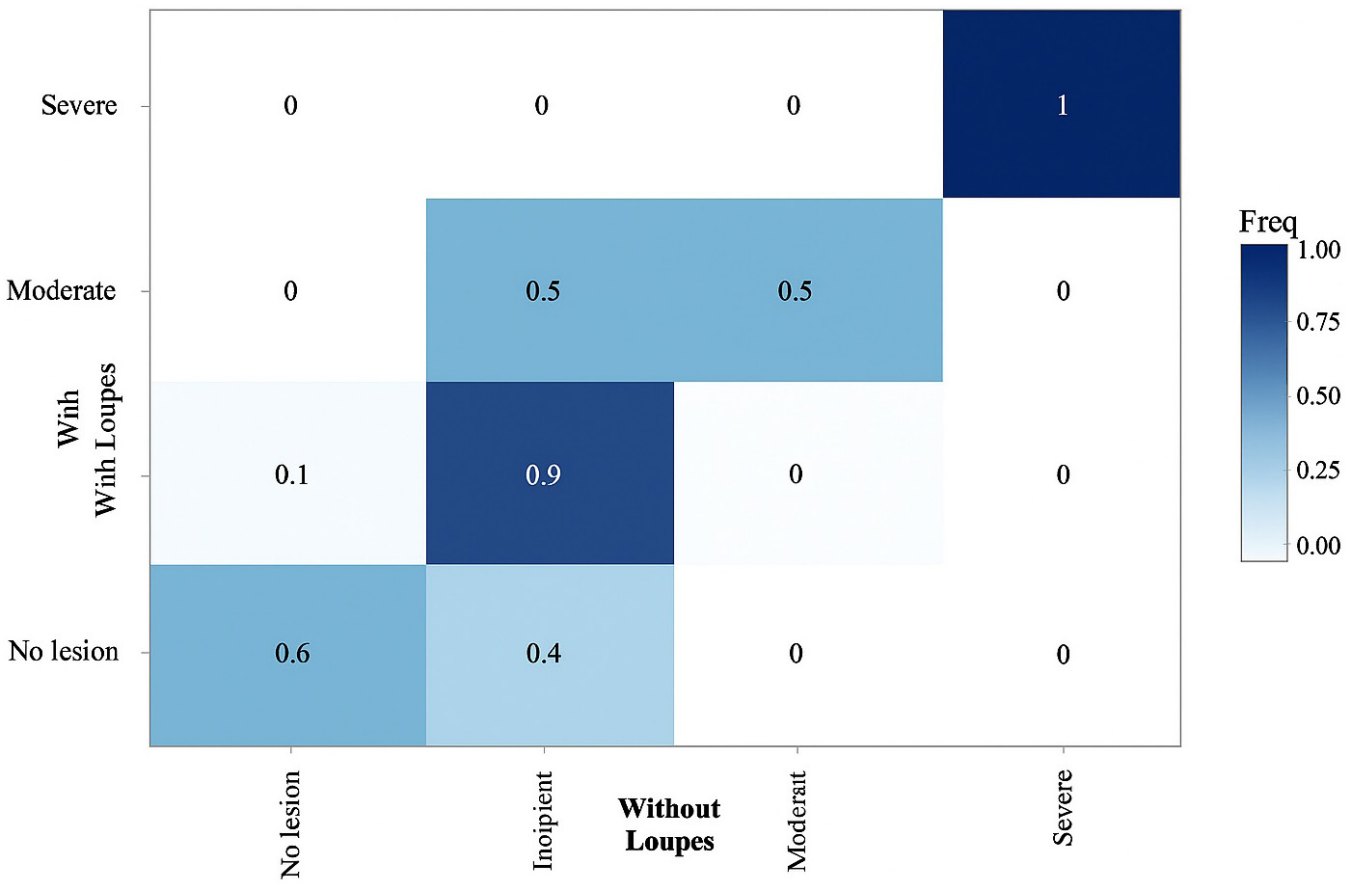
Confusion Matrix.

The concordance analysis aimed to determine the adequacy of the diagnosis made with loupes, using the diagnosis made without loupes as a reference. For each of the methods, the agreement across different days, according to the weighted Kappa coefficient, was high: with loupes: 0.88 [95% CI: 0.88 - 0.88]; without loupes: 0.81 [95% CI: 0.81 - 0.81]. The overall weighted Kappa coefficient was 0.56 (95% CI: 0.43 - 0.69). Additionally, measurements with loupes showed an overall accuracy with respect to measurements without loupes of 0.85 (95% CI: 0.8 - 0.89; shown in Fig. 3).

3

**Figure 4:**
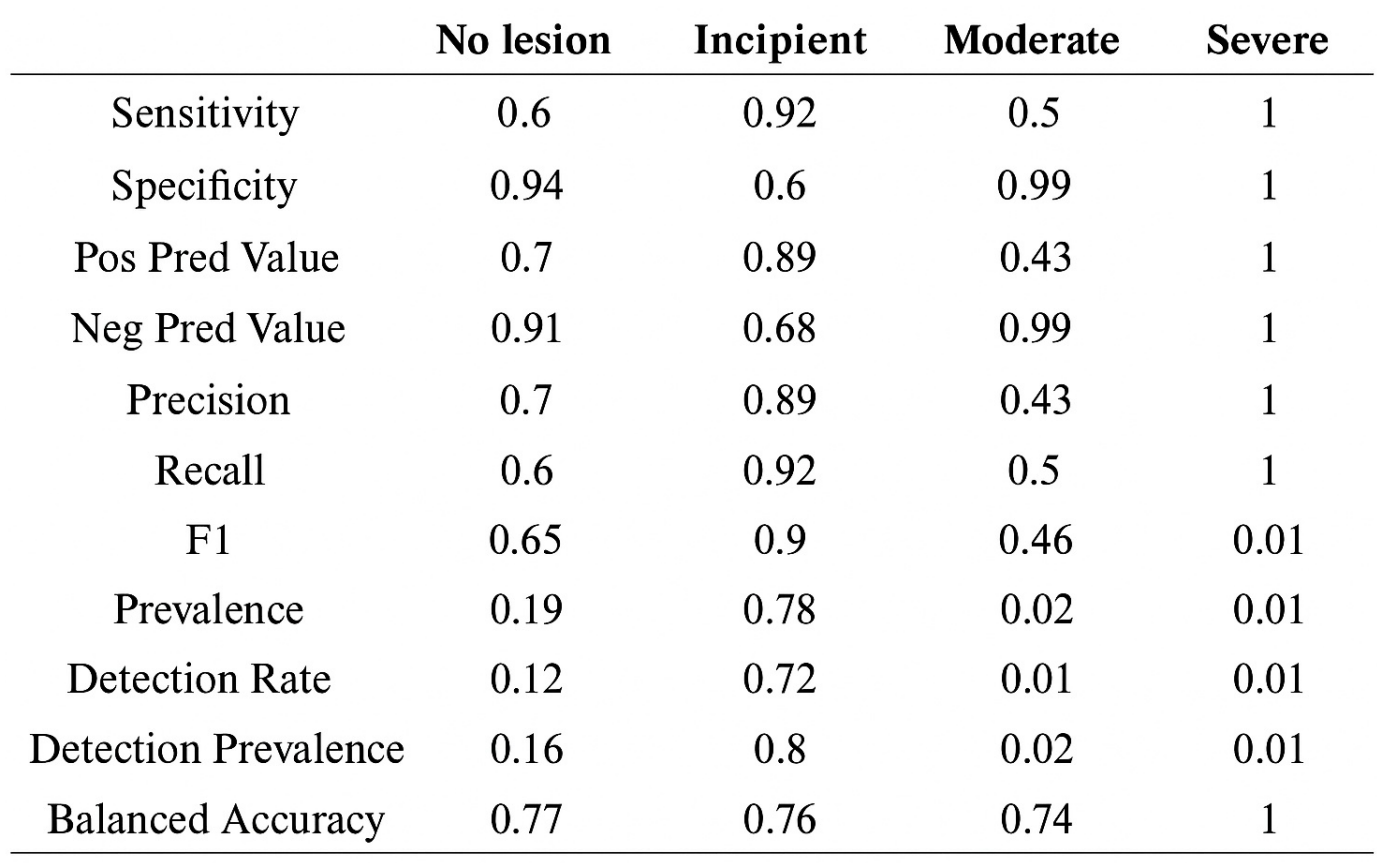
Diagnostic Performance.

The performance metrics for measurements with loupes generally show good sensitivity and specificity values, with balanced accuracy close to 0.75 across all classes (shown in Fig. 4). It should be noted that the "Moderate Lesion" and "Severe Lesion" classes have very low prevalence, which may bias the results for these two categories.

4

## Discussion

These results indicate that the diagnostic method with loupes has moderate agreement with the reference method, suggesting that it may be a useful tool for diagnosing caries in a clinical setting. Moreover, the high Kappa coefficient between measurements taken on different days shows that both methods are reproducible. Thus, according to this, the diagnostic method for caries lesions with loupes is particularly reliable for detecting incipient lesions, which make up the majority of cases in the sample, with very high sensitivity and specificity for the "Severe Lesion" class, although the low prevalence of this class limits the interpretation of these results. For the "No Lesion" class, sensitivity is 0.60 and specificity is 0.94, indicating that the method with loupes is moderately good for detecting caries-free teeth, though there is still room for improvement in sensitivity. The balanced accuracy is 0.77, suggesting a reasonable performance in correctly identifying healthy teeth. In the case of the "Incipient Lesion" class, high sensitivity of 0.92 and specificity of 0.60 are observed. This means that the method with loupes is very effective at correctly detecting incipient lesions, although the ability to rule out other types of lesions is more limited. The balanced accuracy for this class is 0.76. For the "Moderate Lesion" and "Severe Lesion" classes, although sensitivity and specificity values are high, the low prevalence in the sample may influence the stability of these estimates. Specifically, the perfect specificity and sensitivity for "Severe Lesion" reflect the absence of false positives and false negatives in the current sample, but more research with larger samples and higher prevalence of these classes is needed to confirm these findings. In conclusion, the method with loupes shows good overall agreement with the reference method and high accuracy, especially for incipient and severe lesions. However, it is important to consider the low prevalence of moderate and severe lesions when interpreting the results. This analysis suggests that the use of loupes could be a valuable tool in caries detection, though additional studies are recommended to validate these results in larger and more diverse populations, ideally performing accuracy studies of this method in vivo. The early detection of incipient carious lesions is crucial in the prevention and management of dental caries. Identifying these lesions at an early stage allows for the implementation of non-invasive strategies aimed at arresting their progression and even promoting remineralization. Moreover, it provides an opportunity to better control the etiological factors contributing to the development of caries, such as poor oral hygiene, dietary habits, and the presence of cariogenic bacteria. By addressing these underlying causes, it becomes possible to not only treat the current lesions but also reduce the risk of future caries development, improving long-term oral health outcomes. A minimum interval of seven days was decided between all measurements to increase internal validity, as performing measurements with and without loupes at the same time risks biasing the results. Only one (10) of the five studies (10 - 14) with statistically significant positive results on the use of loupes conducted the measurements on different days. No in vivo study with measurements taken on different days was found to show favorable results for the use of loupes (15 - 17). Therefore, a possible line of research is suggested where in vivo diagnosis with and without loupes, as well as radiographic diagnosis, is performed on teeth requiring extraction, followed by histopathological analysis of these extracted teeth to determine the diagnostic accuracy of caries lesions using dental loupes.

## Conclusions

Magnification loupes offer diagnostic accuracy comparable to traditional visual inspection, with greater sensitivity for detecting early carious lesions. Their use may enhance diagnostic reliability and should be considered a valuable adjunct in preventive and minimally invasive dentistry.
